# M6A demethylase ALKBH5 mediated Igfbp4 mRNA m6A modification drives fibroblast activation and pathological upper airway fibrosis

**DOI:** 10.1002/ctm2.70656

**Published:** 2026-04-21

**Authors:** Jing Wang, Ziwei Liao, Mengrou Xu, Yangyang Zheng, Bin Hu, Qianhui Xia, Hongming Xu

**Affiliations:** ^1^ Department of Otorhinolaryngology Head and Neck Surgery, Shanghai Children's Hospital Shanghai Jiao Tong University School of Medicine Shanghai People's Republic of China; ^2^ ENT Institute and Department of Otorhinolaryngology Eye & ENT Hospital Fudan University Shanghai People's Republic of China

**Keywords:** airway fibrosis, ALKBH5, IGFBP4, laryngotracheal, m6A

## Abstract

**Background:**

Laryngotracheal stenosis (LTS) is a fibroproliferative disease of the upper airway characterised by dysregulated extracellular matrix deposition and fibroblast activation. AlkB Homologue 5 (ALKBH5) has emerged as a key regulator of disease pathogenesis by modulating mRNA stability, yet its role in LTS remains unclear.

**Methods:**

We established a LTS rat model and observed mRNA m6A methylation and m6A demethylase ALKBH5 in stenotic tissues. Using single‐cell RNA sequencing, in vivo ALKBH5 knockout models, and in vitro gain‐ and loss‐of‐function assays, we explored the role of ALKBH5 in fibroblast activation and fibrosis. The downstream target of ALKBH5 and its underlying molecular mechanism were identified through integrating RNA‐seq and MeRIP‐seq analyses, and further validated by RNA immunoprecipitation (RIP)‐quantitative PCR (qPCR), dual‐luciferase reporter assays, and in vitro gain‐ and loss‐of‐function experiments. In addition, the therapeutic potential of exogenous modulation of ALKBH5 target was evaluated in LTS models.

**Results:**

ALKBH5 acted as a key pro‐fibrotic regulator by enhancing the expression of COL1A1, COL3A1 and alpha‐smooth muscle actin (α‐SMA), increasing fibroblast contractility, and promoting airway fibrosis progression in LTS. RNA‐seq and MeRIP‐seq analyses identified *Igfbp4* as a direct target of ALKBH5. RIP‐qPCR and luciferase reporter assay confirmed ALKBH5 binding to the 3′‐untranslated region of *Igfbp4*. Functional studies revealed that IGFBP4 inhibited β‐catenin signalling and attenuated fibroblast activation. Overexpression of IGFBP4 partly reversed the profibrotic effects of ALKBH5, both in vitro and in vivo, significantly reducing collagen deposition and airway narrowing in LTS rats.

**Conclusion:**

Our findings identify a novel ALKBH5–IGFBP4 regulatory axis that drives fibroblast activation and airway fibrosis in LTS. Targeting ALKBH5 or supplementing IGFBP4 may become novel therapeutic strategies for LTS.

## Graphical abstract

ALKBH5‐mediated m6A modification of Igfbp4 mRNA drives fibroblast activation and pathological collagen deposition in upper airway.

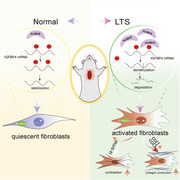

## 1 BACKGROUND

Laryngotracheal stenosis (LTS) is a pathological condition featured by tissue fibrosis in upper airway, which leading to narrowing or atresia of airway lumen and impairing respiratory functions.^1^, ^2^ However, the underlying mechanism of LTS remains unclear. Dysregulated fibroblast activation and abnormal accumulation of collagen and other extracellular matrix (ECM) components are considered hallmarks of fibrosis.^3^ Multiple factors can contribute to fibrosis, including activation of the transforming growth factor‐beta (TGF‐β) signalling pathway,^4^ local ECM niche alterations,^3^ mechanical cues^5^ and metabolic reprogramming.^6^ In recent years, RNA m6A modification has been found to play a regulatory role in fibrosis.

RNA m6A modification regulates the transcriptional profiles of cells by controlling mRNA stability at the post‐transcriptional level, thereby influencing diverse biological functions.^7^ AlkB Homologue 5 (ALKBH5), a key m6A demethylase, removes methyl groups from RNA, effectively reversing m6A modifications. ALKBH5 is involved in various disease processes, including fibrotic disorders,^8^ cancer^9^, ^10^ and endocrine system diseases.^11^ For example, under conditions of acute mycardial ischaemia and hypoxia, ALKBH5 can modulate cardiac fibrosis through various mechanisms.^12^, ^13^ In the repair phase following myocardial infarction, ALKBH5 mediated ErbB4 mRNA stability via m6A demethylation, promoting fibroblasts activation and thus facilitating fibrotic repair.^14^ Overexpression of ALKBH5 had also been shown to upregulate the expression of renal epithelial adhesion molecule E‐cadherin, leading renal fibrosis.^15^ However, conflicting findings have also been reported. In a renal injury model, knockdown of ALKBH5 enhanced the ErbB4 mRNA stability through reversing the m6A modification, which in turn alleviated acute kidney injury and renal fibrosis.^16^ Collectively, these studies indicate that ALKBH5 appears to have context‐dependent roles in tissue fibrosis. These divergent outcomes may be attributed to its distinct functions at different pathological stages or in different cell types.

In this study, we evaluated the regulatory role of ALKBH5 in fibrosis and fibroblast function in the context of LTS. We found that in the LTS animal model, m6A modification levels were decreased, while ALKBH5 expression was upregulated. In vitro, inhibition of ALKBH5 in fibroblasts suppressed the expression of fibrosis‐related proteins, such as α‐SMA and COL1A1. In ALKBH5 knockout (KO) rats, fibrotic remodelling was attenuated and the severity of LTS was alleviated. Furthermore, we identified a potential mechanism whereby ALKBH5 promotes LTS‐associated fibrosis by partly reducing m6A methylation of Igfbp4 mRNA, leading to downregulation of Igfbp4 expression.

## 2 METHODS

### 2.1 ALKBH5 knockout rats

Male ‐ALKBH5 KO (Alkbh5^−^/^−^) Wistar rats aged 12–16 weeks were generated using the CRISPR/Cas9 genome‐editing system. Briefly, two single‐guide RNAs (sgRNAs) were designed to target the intronic regions flanking the Alkbh5 locus. These sgRNAs were synthesised and transcribed in vitro, and subsequently co‐injected with Cas9 protein into zygotes. The injected zygotes were then transferred into females to produce F0 generation offspring. Genotyping of the resulting pups was performed by PCR amplification and Sanger sequencing of genomic DNA extracted from tail biopsies.

### 2.2 LTS model

**FIGURE 1:**
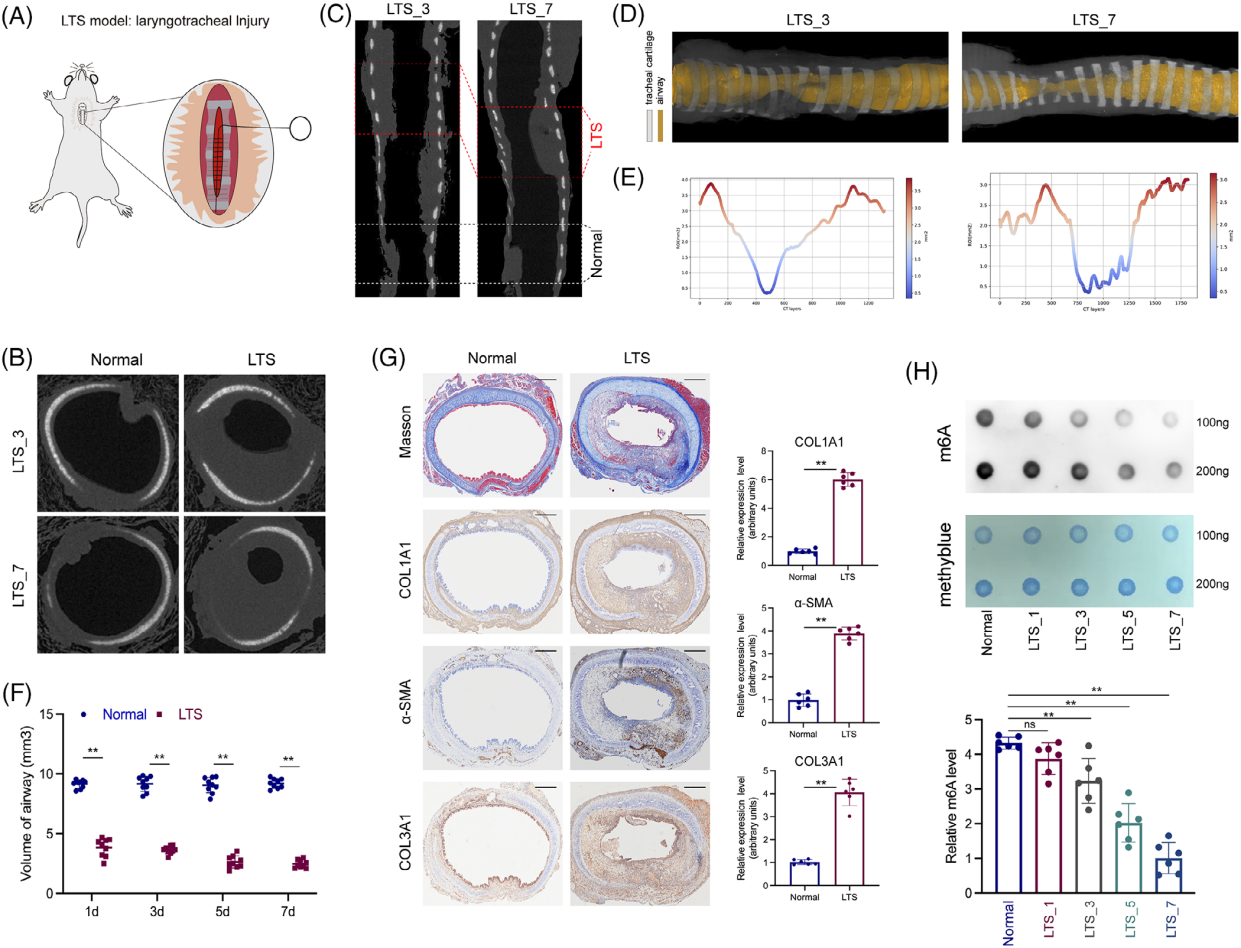
m6A modification was decreased in laryngotracheal stenosis (LTS). (a) The rat model of LTS was induced by injuring the laryngotracheal lining using a brush. (b) Representative images of thin‐section computed tomography cross‐sections of laryngotracheal tissues in rats at 3 and 7 days post‐injury. (c) Representative images of thin‐section computed tomography longitudinal sections of laryngotracheal tissues in rats at 3 and 7 days post‐injury. (d) Representative three‐dimensional reconstructed images of laryngotracheal tissues in rats at 3 and 7 days post‐injury. (e) Quantification of the lumen diameter using regions of interest (ROI) at 3 and 7 days post‐injury. (f) Quantification of the airway volume at days 1, 3, 5 and 7 post‐injury. *N* = 9. (g) Masson's trichrome staining and immunostaining of COL1A1, α‐SMA and COL3A1 of laryngotracheal tissues from rats at normal tissue and 7 days post‐LTS induction. Quantification of the expression levels was shown on the right side. *N* = 6. (h) m6A dot blot analysis of laryngotracheal tissues from normal rats and rats at 1, 3, 5 and 7 days post‐induction. Upper and lower rows indicate 100 and 200 ng of RNA, respectively. Quantification of the expression levels was shown on the below. *N* = 9. Data are presented as mean ± standard deviation (SD); ns: not significant; ^*^ *p* < .05, ^**^ *p* < .01, ^**^ *p* < .001.

Animal experiments were conducted under the approval and supervision of the Institutional Animal Care and Use Committee of Shanghai Children's Hospital. In the initial animal study, 12‐16‐week‐old male rats were subjected to LTS induction. ALKBH5 KO rats and their wild‐type (WT) littermates were used for LTS induction. Igfbp4 adeno‐associated virus (AAV)‐containing gel was placed around the trachea for both the control and experimental groups, as previously described. Three weeks following delivery of adenovirus, rats were subjected to LTS induction. First, intraperitoneal injection of ketamine and xylazine were performed to anaesthetise the rats. A vertical midline incision was made to expose the larynx and trachea after sterilising the skin. A wire brush coated with bleomycin was inserted into the airway and moved back and forth to induce LTS by creating laryngotracheal injury. During this process, full‐thickness injury to the tracheal mucosa was required, exposing the tracheal cartilage to ensure successful LTS formation. Care was taken throughout the procedure to prevent blood from entering the trachea or lungs. After surgical induction, rats were maintained on a high‐protein soft diet. At the beginning, the 7‐day survival rate was approximately 40%–50%. After we became proficient with the surgical procedure, the survival rate could be stable at 80% (the remaining 20% mortality was mainly due to asphyxia during postoperative recovery). In addition, micro‐computed tomography (CT) was performed at tissue collection, showing that the degree of airway stenosis at day 7 was consistently around 70% (Figure 1f), thereby ensuring injury consistency.

### 2.3 Bleomycin‐induced pulmonary fibrosis model

Rats (200–220 g) were divided into control group or bleomycin‐induced fibrosis group randomly. After anaesthesia and secured, rats in the fibrosis group were administered bleomycin (5 mg/kg, dissolved in sterile saline) intratracheally using a microsyringe via oral intubation. Following administration, rats were gently rotated to ensure uniform distribution of the drug within the lungs. Rats were then returned to their cages and monitored closely. On day 35 post‐administration, lung tissues were harvested for subsequent analyses.

### 2.4 Culture of fibroblasts and lentiviral transfection

Fibroblasts from mouse laryngotracheal tissue were cultured in high‐glucose Dulbecco's Modified Eagle Medium (DMEM) supplemented with 10% foetal bovine serum (FBS) and 1% penicillin–streptomycin. Cells were maintained at 37°C in a humidified incubator with 5% CO_2_. Lentiviral vectors for gene knockdown (shAlkbh5, shIgfbp4 and scrambled negative control) and overexpression (Alkbh5, Igfbp4 and empty vector) were generated by Shanghai Zuorun Biotech Ltd. When cell confluence reached 60%–70%, replacing the culture medium with fresh medium containing 5% FBS, and lentivirus was added at a multiplicity of infection of 20. After 12 h, the supernatant was removed and replaced with standard DMEM supplemented with 10% FBS. For selecting stably transduced cells, puromycin (3 µg/mL) was added 48 h post‐infection. Quantitative real‐time PCR (RT‐qPCR) and western blot (WB) analysis were used to evaluate the knockdown and overexpression efficiencies for shAlkbh5, shIgfbp4, Alkbh5 and Igfbp4. The shRNA sequences used are listed in Supporting Information S1.

### 2.5 Immunohistochemical and histology and staining

Rat laryngotracheal tissues were collected and fixed in 4% paraformaldehyde for 24 h. After dehydration in a graded ethanol series and clearing with xylene, tissues were processed paraffin embedding and then sectioned into 5‐µm‐thick slices. These slices were subjected to haematoxylin and eosin staining, Masson's trichrome staining and immunohistochemical (IHC) staining. For IHC, the following primary antibodies were used: anti‐α‐SMA (ab7817, Abcam), anti‐COL1A1 (72026S, Cell Signalling Technology), anti‐ALKBH5 (ab195377, Abcam), anti‐IGFBP4 (ab239594, Abcam) and anti‐COL3A1 (ab7778, Abcam). Slide scanner was used to digitise stained sections, and quantification was carried out using ImageJ software.

### 2.6 Micro‐CT and measurement

Before scanned, tracheal tissues were collected and fixed in 4% paraformaldehyde for 24 h. Micro‐CT imaging was performed using a benchtop CT scanner (nanoVoxel‐2000, Sanying Precision Instruments). The scanning, reconstruction and analysis were carried out using NanoVoxel Scan (v2.1.702.0), VoxelStudio Recon (v2.5.1.25) and CT Vox software (Bruker, v3.2), respectively. After scanning, voxels with grayscale values below 10 000 were defined as air. Tracheal structure reconstruction was then performed. The tracheal inlet and base were identified from the vertical axis view, and the airway region was defined as the enclosed space between these two landmarks. Air within this region was segmented and visualised in yellow. To enhance the visualisation of the airway morphology, the opacity of the surrounding soft tissue was reduced, thereby allowing for clear delineation of the luminal contour.

### 2.7 Quantitative real‐time PCR

Total RNA of tissues or cells was extracted by using TRIzol reagent, according to the manufacturer's instructions and the extracted RNA was reverse transcribed into cDNA using PrimeScript RT Reagent Kit with gDNA Eraser. Then, the cDNA was used to performed RT‐qPCR. Real‐time fluorescence quantitative PCR system was used to quantised gene expression. GAPDH served as the reference gene and the primers are listed in Supporting Information S1.

### 2.8 m6A dot blot

Extracted total RNA was adjusted to a concentration of 100 and 200 ng/µL. An amount of 2 µL RNA was spotted onto a nylon membrane and crosslinked using ultraviolet (UV) light. After stained with methylene blue (MB) for 5 min to visualise total RNA loading, the membrane was photographed, washed, blocked and then incubated overnight at 4°C with a rabbit anti‐m6A primary antibody. The next day, after washed with Phosphate Buffered Saline with Tween (PBST), the membrane was incubated with secondary antibody. Following additional PBST washes, chemiluminescent detection was performed using the Immobilon Western substrate. For the dot blot assays, the quantification was performed using ImageJ based on the grayscale intensity of each dot. Briefly, the integrated density of the m6A signal was measured after background subtraction. To ensure equal RNA loading across samples, the membranes were subsequently stained with MB (loading control), and the corresponding total RNA signal was quantified in the same manner. The final relative m6A level was calculated as the ratio of m6A signal to MB signal (m6A/MB) and was then normalised to the control group, which was set to 1.

### 2.9 MeRIP‐seq and RNA‐seq

Total RNA was extracted, and its quality and concentration were assessed. Following quality control, RNA was fragmented into 100–200 nucleotide (nt) fragments and 100 ng of fragmented RNA was reserved as the input control. The remaining fragmented RNA was incubated in immunoprecipitation (MeRIP) buffer containing anti‐m6A antibody at 4°C for 120 min. The mixture was then incubated with Protein A/G magnetic beads, and m6A‐containing RNA fragments were eluted using an m6A‐specific elution buffer. Following purification, sequencing libraries were constructed using the eluted m6A‐enriched fragments (IP) as well as the input control. Paired‐end sequencing (2 × 150 bp) was performed on an Illumina NovaSeq 6000 platform by LC‐BIO Biotech Ltd. Subsequent data processing included genome alignment, peak calling, transcript annotation and functional enrichment analysis. The quality of m6A‐seq data was rigorously evaluated prior to downstream analysis. MeRIP‐seq and RNA‐seq data information: mouse fibroblasts with shRNA‐mediated ALKBH5 knockdown, eight samples, Sequence Read Archive (SRA): PRJNA1311743 (https://www.ncbi.nlm.nih.gov/sra/PRJNA1311743)

### 2.10 RNA decay assay

RNA decay assay was performed to evaluate the RNA stability. When the cell confluence reached 60%–70%, each well were treated with actinomycin D (5 µg/mL) to block transcription. Cells were collected after 0, 2, 6 and 8 h, respectively, to extracted total RNA. The mRNA levels of target genes at different times were quantified by RT‐qPCR with 0 h as the baseline. The half‐life of the RNA was analysed by PrismGraphPad9.0 (GraphPad Software, Inc.).

### 2.11 RNA immunoprecipitation‐PCR

RNA immunoprecipitation (RIP)‐PCR was performed to validate the interaction between ALKBH5 and Igfbp4 mRNA. Cells were cross‐linked with  .75% formaldehyde for 10 min at room temperature and quenched with  .125 M glycine. Cells were subsequently lysed in RIP lysis buffer supplemented with RNase inhibitors (Promega, N2611) and protease inhibitors (Roche, 12352204). The lysates were then sonicated (25 amplitude with 15 cycles of 10 s ON and 10 s OFF on ice) to shear RNA to an average fragment length of 300–500 nt. After centrifugation, the supernatants were incubated with anti‐ALKBH5 antibody (Proteintech, Cat# 16837‐1‐AP) or control immunoglobulin for 4 h at 4°C, followed by capture with Protein A/G magnetic beads. The beads were then washed and treated with Proteinase K at 55°C to digest proteins and incubated at 65°C to reverse cross‐links. Finally, co‐precipitated RNA was extracted using Trizol reagent and analysed by RT‐qPCR. qPCR was performed using SYBR Green Master Mix with specific primers targeting Igfbp4‐binding sites (sites 1–3) and internal positive control HECBPA (hepatocyte nuclear factor 4 alpha‐binding protein). The primers are listed in Supporting Information S1. The reaction was run at 95°C for 3 min, followed by 40 cycles of 95°C for 10 s and 60°C for 30 s, with melt curve analysis. Relative enrichment was calculated using the 2^^−ΔΔCt^ method. PCR products were validated by agarose gel electrophoresis.

### 2.12 Western blot

For sample preparation, tissues were minced and lysed in RIPA lysis buffer supplemented with protease and phosphatase inhibitors. After complete lysis, samples were subjected to centrifuge. The supernatants were collected, mixed with loading buffer and then boiled at 98°C for 5 min to denature proteins. The bicinchoninic acid assay (Thermo Fisher Scientific) were perform to tested the concentrations of protein. Then, 20 µg protein samples were loaded into separate lanes of SDS‐PAGE gels for electrophoresis and transferred onto methanol‐activated Polyvinylidene Fluoride (PVDF) membranes using a wet transfer method. After blocked with 5% non‐fat milk in Tris‐Buffered Saline with Tween‐20 (TBST), the membranes were incubated in appropriate primary antibodies overnight at 4°C. The next day, after washed with PBST, the membrane was incubated with secondary antibody. Enhanced Chemiluminescence (ECL) WB detection system was used to detected chemiluminescent signals, and band intensities were quantified by grayscale analysis. The primary antibodies are listed in Supporting Information S1.

### 2.13 Dual‐luciferase reporter assay

To assess the regulatory effect of ALKBH5 on sites 1–3, a dual‐luciferase reporter assay was conducted. HEK293T cells were cultured in DMEM supplemented with 10% FBS at 37°C and 5% CO_2_. *Escherichia coli* was cultured in LB medium and stored in 20% glycerol at −80°C. The luciferase reporter constructs were generated by amplifying target sequences (sites 1–3) via PCR using Phanta Max Master Mix, and cloned into the pcDNA3.1(+)‐LUC vector. ALKBH5 overexpression plasmids were constructed in pcDNA3.1(+). Recombinant plasmids were transformed into *E. coli*, and plasmid DNA was extracted using an endotoxin‐free Maxi prep kit (Thermo, A33073). For transfection, 293T cells at ∼80% confluence were washed with phosphate‐buffered saline (PBS) and transfected with the constructed plasmids using Lipofectamine 2000 according to the manufacturer's protocol. After 4 h incubation, the medium was replaced with complete DMEM and cells were cultured for an additional 24 h. The dual‐luciferase reporter assay was performed using the Dual Luciferase Reporter Assay Kit (Vazyme, DL101‐01). Cells were lysed in 1× cell lysis buffer, and 20 µL of lysate was transferred into a 96‐well plate. Firefly luciferase (LUC) activity was measured first by adding 100 µL of LUC substrate, followed by Renilla luciferase (RLUC) measurement using the Stop & Glo reagent. Luminescence was measured using a microplate reader (BioTek), and relative reporter activity was calculated as the ratio of LUC to RLUC.

### 2.14 Single‐cell RNA sequencing analysis

Laryngotracheal tissues were harvested from rats (*n* = 3 per group) at 1, 3, 5 and 7 days post‐induction. Samples were cut into 1 mm^3^ fragments and digested, and filtered through 40‐µm strainers. After PBS washing (800×*g*, 5 min) and viability confirmation (>80% by Trypan Blue exclusion), single‐cell suspensions in PBS/1% FBS were processed using the 10× Genomics Chromium platform (LC‐Bio). The detailed single‐cell RNA sequencing (scRNA‐seq) procedures and data analysis methods are described in our previous publication.^17^

Bioinformatic analysis utilised Seurat (v4.3.0). Quality control excluded cells with <500 detected genes or >25% mitochondrial content, retaining 47 068 cells. Data underwent library size normalisation and identification of top 2000 variable genes (flavor = ‘Seurat’). Principal component analysis reduced dimensionality (top 50 PCs), enabling graph‐based clustering (30 clusters) visualised via Uniform Manifold Approximation and Projection (UMAP)/t‐distributed Stochastic Neighbor Embedding (t‐SNE). Differentially expressed genes underwent Gene Ontology (GO) and Kyoto Encyclopedia of Genes and Genomes (KEGG) enrichment analyses using clusterProfiler, with significant terms (*p* < .05) visualised via ggplot2. Differences between two groups were evaluated with Student's *t*‐test, while one‐way analysis of variance (ANOVA) was applied for comparisons among multiple groups, with significance at *p* < .05. Rat laryngotracheal tissues scRNA‐seq data information: rat laryngotracheal tissues, 12 samples, SRA: PRJNA1221101 (https://www.ncbi.nlm.nih.gov/sra/PRJNA1221101); mouse lung scRNA‐seq: three samples, GEO GSE250396 (https://www.ncbi.nlm.nih.gov/geo/query/acc.cgi?acc=GSE250396).

### 2.15 Pearson correlation coefficient matrix

The m6A regulatory enzyme genes (Mettl3, Alkbh5, etc.) and fibrosis‐related genes (Col1a1, Acta2, etc.) were extracted and normalised using DESeq2. Corrplot R package was used to calculated the Pearson correlation coefficient matrix between genes. Significance testing was performed, followed by false discovery rate correction via the Benjamini‒Hochberg method (significance threshold: adjusted *p*‐value <.05). The result was visualised by hierarchical clustering heatmaps with both rows and columns clustered by the Ward's D2 method.

### 2.16 Statistical analysis

Statistical analyses were conducted using GraphPad prism version 10.0 and SPSS version 20.0. Intergroup differences were assessed through independent‐samples *t*‐tests for pairwise comparisons. Multiple group analyses employed one‐way ANOVA with Tukey's post hoc testing. Continuous variables following normal distribution are reported as means ± standard deviation, while non‐normally distributed data appear as medians with ranges. A significance threshold of *p* < .05 was established for all inferential tests. Experimental procedures were replicated in triplicate minimum.

## 3 RESULTS

### 3.1 m6A modification was decreased in LTS

To investigate the changes in m6A modification and its regulatory enzymes during the LTS progression, we performed LTS‐induction in 12‐week‐old male rats (Figure 1a). After induction, the rats exhibited characteristic physiological manifestations of LTS, including increased respiratory rates and inspiratory stridor (Auxiliary Supporting Information). To assess the features of stenosis, we performed micro‐CT scans on days 3 and 7 post‐injury. Thin‐section CT imaging revealed that, by day 3, the tracheal inner lining within the stenotic region was markedly thickened compared with adjacent normal areas (Figure 1b,c). In addition, we employed three‐dimensional (3D) CT reconstruction to evaluate changes in intratracheal volume. Morphological assessment demonstrated a significant reduction in luminal space, with the inner diameter of the stenotic segment being substantially narrower than that of the unaffected regions (Figure 1d,e). Quantitative analysis further confirmed these findings (Figure 1f). These results demonstrated that we successfully established a reproducible model of LTS in rats.^17^, ^18^, ^19^ Then, we performed histological and IHC analyses. Masson's trichrome staining revealed that, concomitant with luminal narrowing, collagen deposition was markedly increased in the stenotic regions compared with normal controls (Figure 1g). IHC staining demonstrated elevated expression of fibrosis‐related markers, including COL1A1, COL3A1 and α‐SMA, in the stenotic areas (Figure 1g). The result were quantitatively analysed. To investigate whether m6A modification was altered during the progression of LTS, we measured global m6A levels in total RNA extracted from normal tracheal tissues and from tracheal tissues at 1, 3, 5 and 7 days post‐injury. We observed a gradual decrease in m6A levels over time, with a sharp drop at days 5 and 7 compared with earlier time points (Figure 1h), and statistical analysis further confirmed these changes. Together, we verified that fibrosis and alterations in m6A modification occurred in LTS.

### 3.2 ALKBH5 expression was increased during the progression of LTS

**FIGURE 2:**
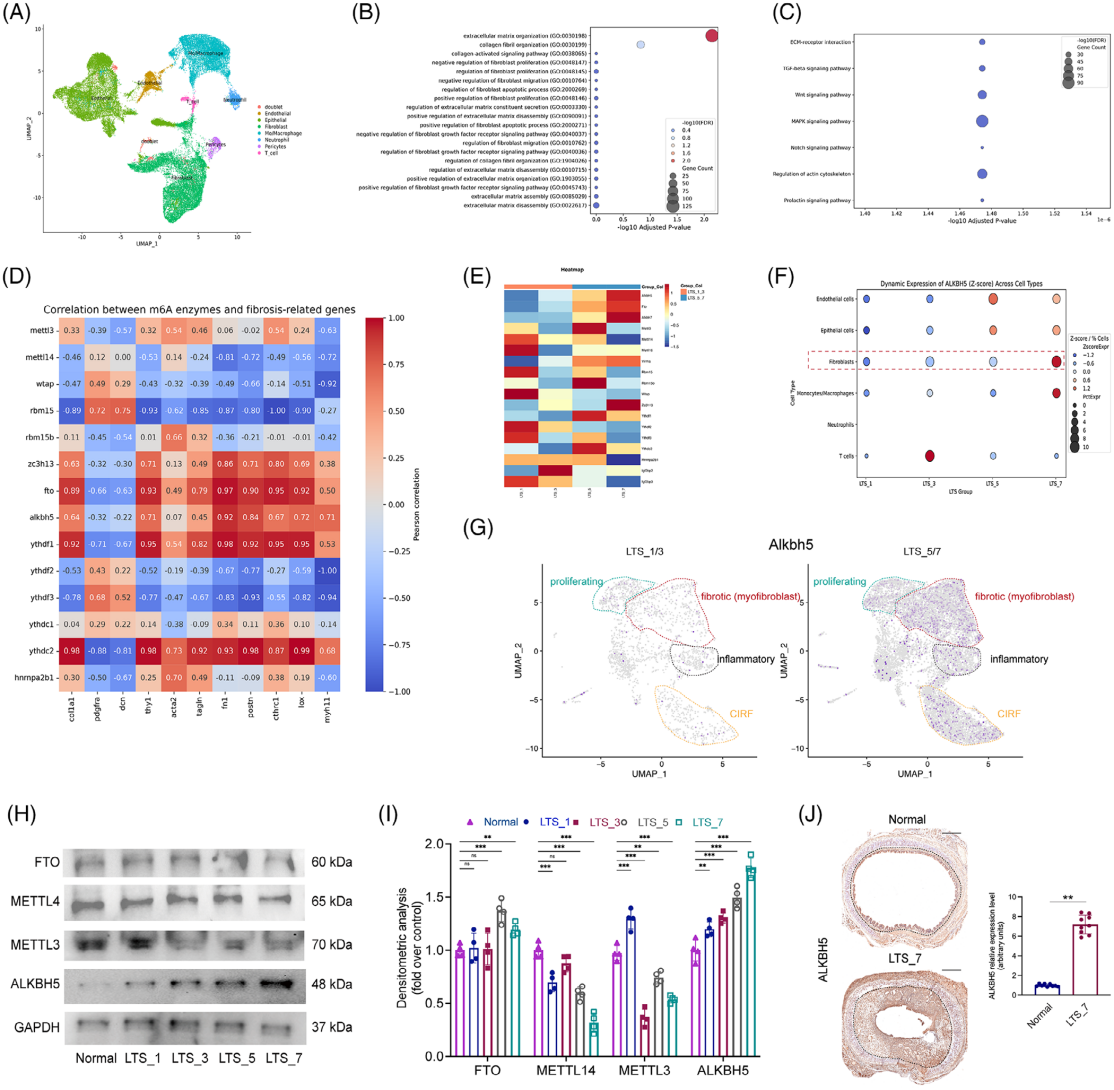
AlkB Homologue 5 (ALKBH5) expression was elevated during the progression of laryngotracheal stenosis (LTS). (a) Manifold Approximation and Projection (UMAP) depicting major eight cell types identified during LTS progression. (b) Gene Ontology (GO) enrichment scatterplot displayed the top 20 significantly enriched biological processes based on upregulated genes identified in fibroblast. (c) Kyoto Encyclopedia of Genes and Genomes (KEGG) enrichment scatterplot illustrated the enriched pathways based on upregulated genes in fibroblast. (d) Heatmap showed the correlation between m6A enzymes and fibrosis‐related genes, with the colour representing the Pearson correlation coefficient, ranging from low (blue) to high (red). (e) Heatmap showed the expression level of m6A modification enzymes at different stages post‐injury. (f) Scatterplot displayed the dynamic ALKBH5 expression in different cell types. (g) UMAP plot depicted the expression of ALKBH5 in distinct fibroblast subpopulations identified during LTS progression, with colour intensity reflecting expression levels, ranging from low to high. Cells from days 1 and 3 after LTS induction were represented in the left panel, while cells from days 5 and 7 were showed in the right one. (h) Western blot showed the expression of FTO, METTL3, METTL14, ALKBH5 and GAPDH in laryngotracheal tissues from normal rats and rats at 1, 3, 5 and 7 days post‐induction. The expression levels were quantified on figure (i). *N* = 4. (j) Representative images of immunostaining of ALKBH5 of laryngotracheal tissues, and the expression levels were quantified on the right side. Data are presented as mean ± standard deviation (SD); ns: not significant; ^*^ *p* < .05, ^**^ *p* < .01, ^**^ *p* < .001. Single‐cell RNA sequencing (scRNA‐seq) data information: rat laryngotracheal tissues, 12 samples, Sequence Read Archive (SRA): PRJNA1221101.

To investigate which m6A modification enzymes may induce the altered m6A modification observed in LTS, we analysed our single‐cell transcriptomic data of LTS rat models^17^ (Figure 2a). After quality control of the scRNA‐seq data, fibroblasts were identified and extracted for downstream analysis. GO enrichment revealed significant activation of fibrosis‐related biological processes, including ‘ECM remodelling’ and ‘collagen fiber remodelling’ (Figure 2b). KEGG pathway analysis showed significant enrichment of fibrosis‐associated signalling pathways, such as the ‘ECM‒receptor interaction’ and ‘TGF‐β signalling pathway’ (Figure 2c). Pearson correlation analysis demonstrated that Alkbh5, Fto and Ythdf1 were positively correlated with Col1a1 (Figure 2d). Heatmap visualisation revealed that Alkbh5 was markedly upregulated at days 5 and 7 post‐injury (Figure 2e). UMAP analysis of the scRNA‐seq data also revealed that Alkbh5 expression was markedly enriched at days 5 and 7 post‐injury (Figure S4a). Notably, Alkbh5 expression was predominantly elevated in fibroblasts and showed a significant increase at day 7 post‐injury (Figure 2f). Together, these single‐cell transcriptomic findings suggested that ALKBH5 may be a main contributor to the changes in m6A modification during LTS progression.

We further investigated the expression dynamics of ALKBH5 at different fibroblasts populations. We performed unsupervised clustering on fibroblasts identified from our single‐cell RNA‐seq data, and UMAP analysis revealed that Alkbh5 expression was markedly enriched in the fibrotic fibroblast subset at days 5 and 7 post‐injury (Figure 2g). The details of clustering was described in our previous publication.^17^ To validate these findings at the protein level, we performed ex vivo WB analysis and confirmed that the expression levels of ALKBH5 protein was significantly increased, with quantification supporting these observations (Figure 2h,i). IHC staining further demonstrated elevated ALKBH5 expression in the stenotic regions of the trachea (Figure 2j). Taken together, these findings were further highlighted a robust upregulation of ALKBH5 in fibroblasts during the LTS process. In addition, we explored scRNA‐seq data (GSE250396) from a mouse model of pulmonary fibrosis (Figure S1a). GO enrichment analysis revealed that samples collected at day 35 post‐induction exhibited significant enrichment in ECM‐related processes. Key fibrotic markers, including Col1a1 and Acta2, were notably upregulated (Figure S1b). Gene Set Enrichment Analysis further confirmed that gene sets involved in collagen fibril organisation and ECM components were significantly enriched in day 35 samples (Figure S1c), indicating the development of pulmonary fibrosis. Importantly, Alkbh5 expression was markedly elevated in these fibrotic samples (Figure S1d). We also established a rat model of pulmonary fibrosis and performed IHC staining on lung tissue samples. The results further confirmed that the expression levels of COL1A1, COL3A1, α‐SMA and ALKBH5 were significantly elevated in fibrotic lung tissues (Figure S2). To investigate potential upstream regulators of ALKBH5, fibroblasts were treated withhypoxia‐inducible factor1‐alpha (HIF‐α)or TGF‐β. We found that ALKBH5 expression was specifically upregulated following TGF‐β treatment (Figure S4c). These results suggest that inflammatory microenvironments may modulate ALKBH5 expression, thereby contributing to fibrosis.

### 3.3 Silencing ALKBH5 ameliorated LTS formation

**FIGURE 3:**
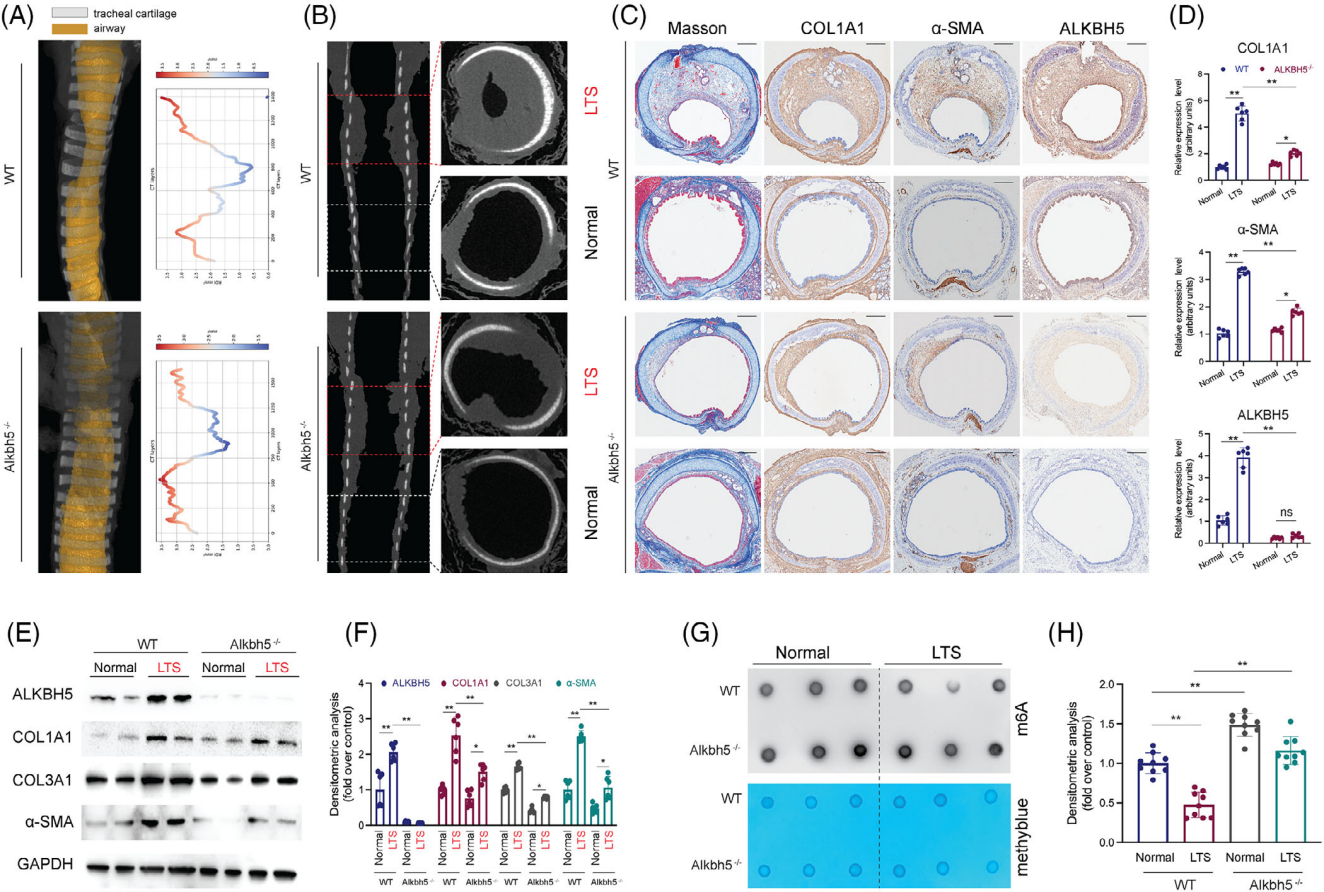
Silencing AlkB Homologue 5 (ALKBH5) ameliorated laryngotracheal stenosis (LTS) formation. (a) Representative three‐dimensional reconstructed images of laryngotracheal tissues in Alkbh5^−^/^−^ rats and wild‐type (WT) rats at 7 days post‐injury. Quantification of the lumen diameter using regions of interest (ROI) was shown on the right. (b) Representative images of thin‐section computed tomography longitudinal sections of laryngotracheal tissues in Alkbh5^−^/^−^ rats and WT rats at 7 days post‐injury. Cross‐sections shown on the right. (c) Masson's trichrome staining and immunostaining of COL1A1, α‐SMA and ALKBH5 of laryngotracheal tissues from Alkbh5^−^/^−^ rats and WT rats with LTS or not, and the expression levels were quantified on figure (d). *N* = 6. (e) Western blot showed the expression of COL1A1, α‐SMA, ALKBH5, COL3A1 and GAPDH in laryngotracheal tissues from Alkbh5^−^/^−^ rats and WT rats with LTS or not. (f) Quantification of the expression levels of western blot. *N* = 6. (g) Representative m6A dot blot analysis of laryngotracheal tissues from Alkbh5^−^/^−^ rats and WT rats with LTS or not. (h) Quantification of the m6A modification levels. *N* = 9. Data are presented as mean ± standard deviation (SD); ns: not significant; ^*^ *p* < .05, ^**^ *p* < .01, ^**^ *p* < .001.

To further evaluate the role of ALKBH5 in the development of LTS in vivo, we generated Alkbh5 KO (Alkbh5^−^/^−^) rats by deleting two exons of the Alkbh5 gene. The KO efficiency was confirmed by WB analysis (Figure 3e). LTS models were subsequently established in both Alkbh5^−^/^−^ and WT rats to elucidate the specific contribution of Alkbh5 to LTS pathogenesis. Three‐dimensional CT imaging revealed that Alkbh5^−^/^−^ rats exhibited less severe airway obstruction compared with their WT littermates (Figure 3a), accompanied by a thinner tracheal mucosa (Figure 3b). Masson's trichrome staining demonstrated a marked reduction in mucosal thickness, luminal narrowing and ECM deposition in Alkbh5^−^/^−^ LTS rats relative to WT controls (Figure 3c). IHC staining further revealed significantly lower expression of COL1A1, α‐SMA and ALKBH5 in the tracheal tissues of Alkbh5^−^/^−^ rats, suggesting that ALKBH5 promotes fibroblast activation. Consistent with these findings, WB analysis of isolated fibroblasts from Alkbh5^−^/^−^ rats showed a significant reduction in COL1A1, COL3A1, α‐SMA and ALKBH5 expression compared with those from WT rats (Figure 3e,f). To assess whether ALKBH5 deficiency affected m6A methylation in vivo, we measured global m6A levels in total RNA extracted from both stenotic and normal tracheal tissues at day 7 post‐injury. Notably, Alkbh5^−^/^−^ rats exhibited significantly increased m6A levels in the stenotic regions, while no substantial changes were observed in the normal areas (Figure 3g). Statistical analysis further validated these observations (Figure 3h). Furthermore, we established a pulmonary fibrosis model in both WT and Alkbh5^−^/^−^ rats. The results showed evident pulmonary fibrosis in WT rats, characterised by collagen deposition (Masson's trichrome staining) and increased expression of COL1A1, α‐SMA and ALKBH5 (IHC staining). In contrast, Alkbh5^−^/^−^ rats exhibited significantly reduced collagen deposition and lower expression levels of COL1A1, α‐SMA and ALKBH5 compared with fibrotic WT rats (Figure S3). In summary, these results demonstrated that ALKBH5 deficiency mitigated LTS formation by suppressing the expression of COL1A1, COL3A1 and α‐SMA, highlighting ALKBH5 as a key pro‐fibrotic factor in the pathogenesis of LTS.

### 3.4 ALKBH5 drived fibroblast activation in vitro

**FIGURE 4:**
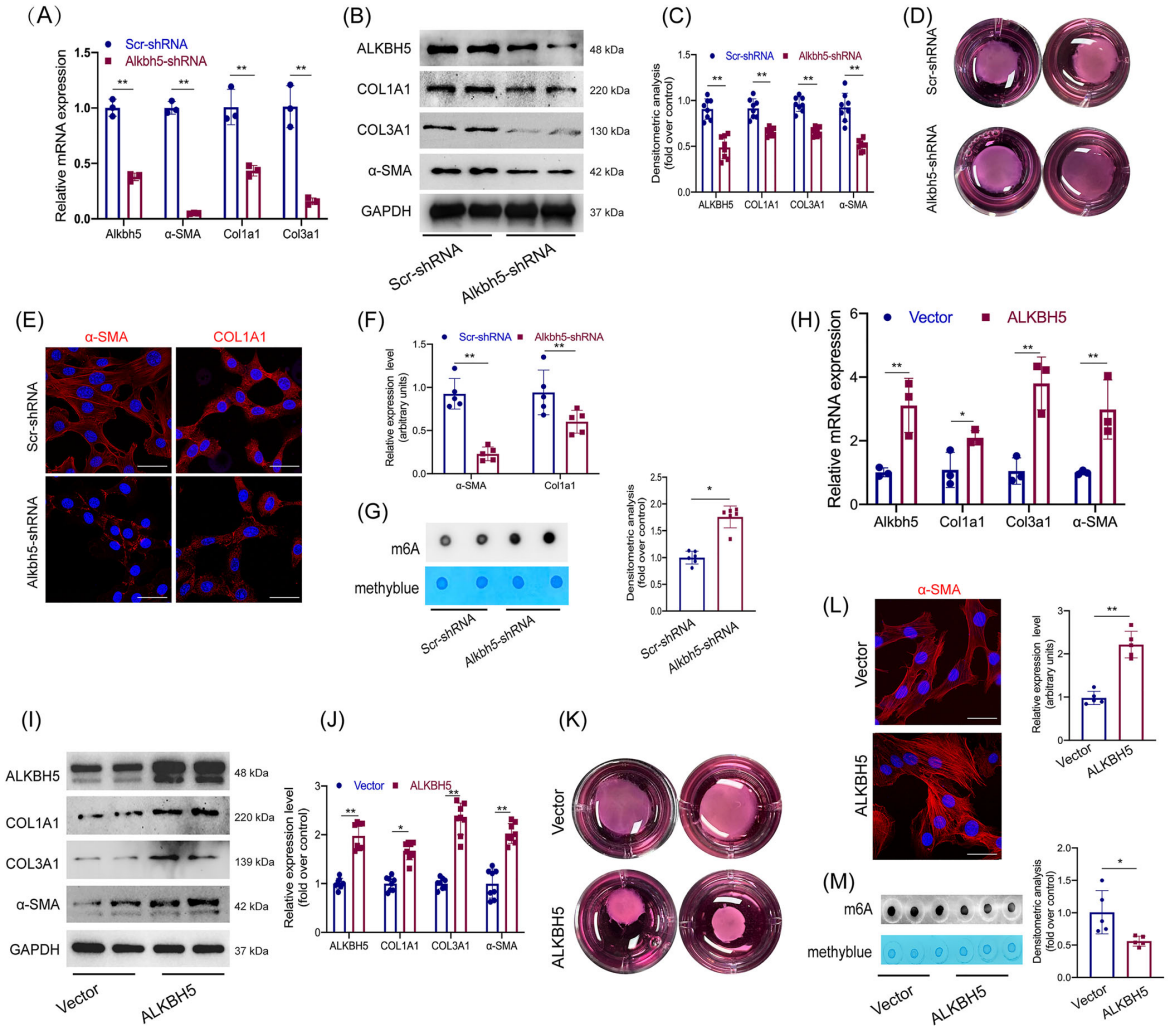
AlkB Homologue 5 (ALKBH5) drive fibroblast activation in vitro. (a) The mRNA expression levels of Alkbh5, α‐Sma, Col1a1 and Col3a1 in cells treated with scr‐shRNA or Alkbh5‐shRNA. *N* = 3. (b) Western blot showed the expression of COL1A1, α‐SMA, ALKBH5, COL3A1 and GAPDH in cells treated with scr‐shRNA or Alkbh5‐shRNA, and the quantification of the expression levels in western blot were shown in (c). *N* = 8. (d) Collagen gel contraction assay showing the contractility of cell treated with scr‐shRNA or Alkbh5‐shRNA. (e) Representative images of immunofluorescence experiment of α‐SMA and COL1A1 in cells treated with scr‐shRNA or Alkbh5‐shRNA, and the quantification of the expression levels in immunofluorescence were shown in (f). *N* = 5. (g) Representative m6A dot blot analysis of cells treated with scr‐shRNA or Alkbh5‐shRNA, and the m6A modification levels were quantified and shown on the right side. *N* = 6. (h) The mRNA expression levels of Alkbh5, α‐Sma Col1a1 and Col3a1 in cells treated with Lv‐vetor or Lv‐Alkbh5. *N* = 3. (i) Western blot showed the expression level of COL1A1, α‐SMA, ALKBH5, COL3A1 and GAPDH in cells treated with Lv‐vetor or Lv‐Alkbh5, and the expression levels were quantified and shown in (j). *N* = 8. (k) Collagen gel contraction assay showing the contractility of cell treated with Lv‐vetor or Lv‐Alkbh5. (l) Representative images of immunofluorescence experiment of α‐SMA in cells treated with Lv‐vetor or Lv‐Alkbh5, and quantification of the expression levels was shown on the right side. (m) Representative m6A dot blot analysis of cells treated with Lv‐vetor or Lv‐Alkbh5, and the m6A modification levels were quantified and shown on the right side. *N* = 5. Data are presented as mean ± standard deviation (SD); ns: not significant; ^*^ *p* < .05, ^**^ *p* < .01, ^**^ *p* < .001.

Although deletion of ALKBH5 reduced collagen deposition and fibroblast activation in vivo, its direct effect on the biological behaviour of laryngotracheal fibroblasts remained unclear. To investigate this in vitro, we isolated fibroblasts from mouse laryngotracheal tissue and constructed Alkbh5‐targeting shRNA to knockdown ALKBH5 expression. Knockdown efficiency was validated by qPCR and WB (Figure 4a,b). Silencing Alkbh5 significantly reduced the expression of fibrotic markers COL1A1, COL3A1 and α‐SMA, as demonstrated by qPCR, WB (Figure 4a–c) and immunofluorescence staining (Figure 4e,f). Furthermore, collagen gel contraction assays revealed that fibroblast contractility was markedly suppressed upon ALKBH5 knockdown (Figure 4d). Dot blot analysis showed that global m6A levels in total RNA were elevated in ALKBH5‐deficient fibroblasts (Figure 4g).

We next generated fibroblasts with ALKBH5 overexpression, and confirmed overexpression efficiency via qPCR and WB (Figure 4h–j). Overexpression of ALKBH5 resulted in increased expression of COL1A1, COL3A1 and α‐SMA (Figure 4h–j), along with enhanced fibroblast contractility in the collagen gel assay (Figure 4k). Immunofluorescence staining further confirmed elevated α‐SMA expression following ALKBH5 overexpression (Figure 4l). In contrast to knockdown results, dot blot analysis revealed reduced global m6A levels in fibroblasts overexpressing ALKBH5 (Figure 4m). We next attempted pharmacological validation of ALKBH5 function using the selective inhibitor DDO‐2728. Treatment with DDO‐2728 mitigated fibrotic phenotypes in fibroblasts, as evidenced by decreased expression levels of key fibrosis markers, including COL1A1, α‐SMA and COL3A1 (Figure S5a). Taken together, these findings indicated that ALKBH5 promoted the expression of fibrotic markers COL1A1, COL3A1 and α‐SMA, and enhanced fibroblast contractility, highlighting its critical role in fibroblast activation.

### 3.5 Igfbp4 was the downstream target of ALKBH5

**FIGURE 5:**
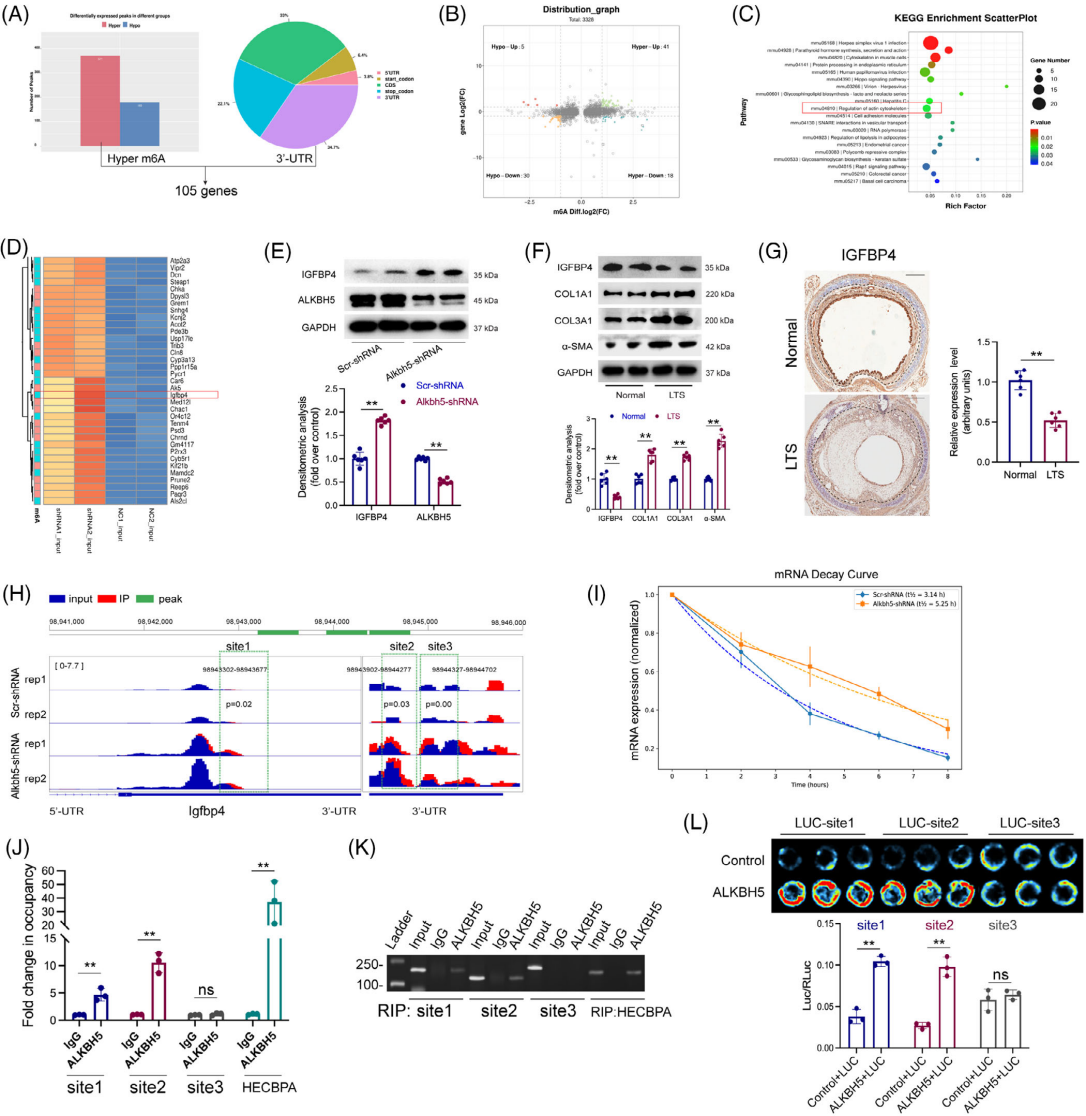
Igfbp4 was the downstream target of AlkB Homologue 5 (ALKBH5). (a) Overlap of genes with increased m6A modification after ALKBH5 knockdown and those with upregulated m6A in 3′‐untranslated regions (UTRs). (b) Volcano plot showed the distribution of genes across m6A change levels in cells with ALKBH5 knockdown compared with control. (c) Kyoto Encyclopedia of Genes and Genomes (KEGG) enrichment scatterplot of enriched pathways based on upregulated genes in cells with ALKBH5 knockdown compared with control. (d) Heatmap of top 30 upregulated gene in cells with ALKBH5 knockdown compared with control. (e) Western blot of IGFBP4 and ALKBH5 in cells treated with scr‐shRNA or ALKBH5‐shRNA, with quantification below. *N* = 6. (f) Western blot of IGFBP4, COL1A1, α‐SMA and COL3A1 in laryngotracheal tissues from rats with laryngotracheal stenosis (LTS) or not, with quantification below. *N* = 6. (g) Immunostaining of IGFBP4 in laryngotracheal tissues from rats with LTS or not, with quantification at right. *N* = 6. (h) Integrative Genomics Viewer plot of m6A modification signals across the Igfbp4 gene locus. The blue background track represented input control signals, and the red track indicated the m6A immunoprecipitation (IP) signals. Three predicted m6A sites boxed. (i) Decay curve of Igfbp4 mRNA after transcription inhibition. (j) RNA immunoprecipitation PCR showed the fold change in ALKBH5 binding at three predicted m6A sites on Igfbp4 mRNA, with HECBPA as a positive control. *N* = 3. (K) Agarose gel electrophoresis showing RNA immunoprecipitation (RIP) results for ALKBH5 occupancy. (l) Dual‐luciferase assays showed the binding of ALKBH5 on the three predicted m6A modification sites constructed in luciferase reporter plasmids. *N* = 3. Data are presented as mean ± standard deviation (SD); ns: not significant; ^*^ *p* < .05, ^**^ *p* < .01, ^**^ *p* < .001. MeRIP‐seq and RNA‐seq data information: mouse fibroblasts with shRNA‐mediated ALKBH5 knockdown, eight samples (knockdown + control), Sequence Read Archive (SRA): PRJNA1311743.

To identify the downstream target of ALKBH5, we conducted RNA‐seq and MeRIP‐seq analyses on ALKBH5 knockdown fibroblasts. We focused on genes that exhibited increased m6A modification in the 3′‐untranslated region (UTR) region, identifying a total of 105 candidate genes (Figure 5a). By integrating the RNA‐seq results, we further narrowed these down to 18 genes that showed both increased m6A modification and elevated expression levels (Figure 5b). KEGG pathway enrichment analysis revealed significant clustering in the regulation of the actin cytoskeleton pathway (Figure 5c). Heatmap analysis highlighted Igfbp4 (insulin‐like growth factor‐binding protein 4) as a potential downstream target of ALKBH5 (Figure 5d). To validate the sequencing data, we performed WB analysis and found that ALKBH5 knockdown led to increased expression of IGFBP4 in fibroblasts (Figure 5e). And ALKBH5 inhibitor DDO‐2728 can also increase the expression levels of IGFBP4 (Figure S5a). LTS tissue samples showed elevated expression of COL1A1, COL3A1 and α‐SMA, while IGFBP4 expression was significantly decreased (Figure 5f). IHC staining further confirmed the downregulation of IGFBP4 in LTS tissues (Figure 5g). To investigate whether ALKBH5 directly binds to Igfbp4 mRNA and to underlie its binding regions, Integrative Genomics Viewer analysis of MeRIP‐seq data identified three putative m6A‐binding sites within the 3′‐UTR of Igfbp4 mRNA (Figure 5h). mRNA stability assays demonstrated that ALKBH5 knockdown significantly prolonged the half‐life of Igfbp4 mRNA (Figure 5i). Using primers specific to the three predicted sites, RIP‐PCR assays demonstrated that ALKBH5 binds to site 1 and site 2, but not site 3 (Figure 5j,k). Furthermore, dual‐luciferase reporter assays confirmed that ALKBH5 protein directly interacted with reporter constructs containing site 1 and site 2 sequences from Igfbp4 3′‐UTR, while no binding was observed at site 3 (Figure 5l). Collectively, these results demonstrated that Igfbp4 is a direct downstream target of ALKBH5.

### 3.6 IGFBP4 inhibited fibroblast activation in vitro

**FIGURE 6:**
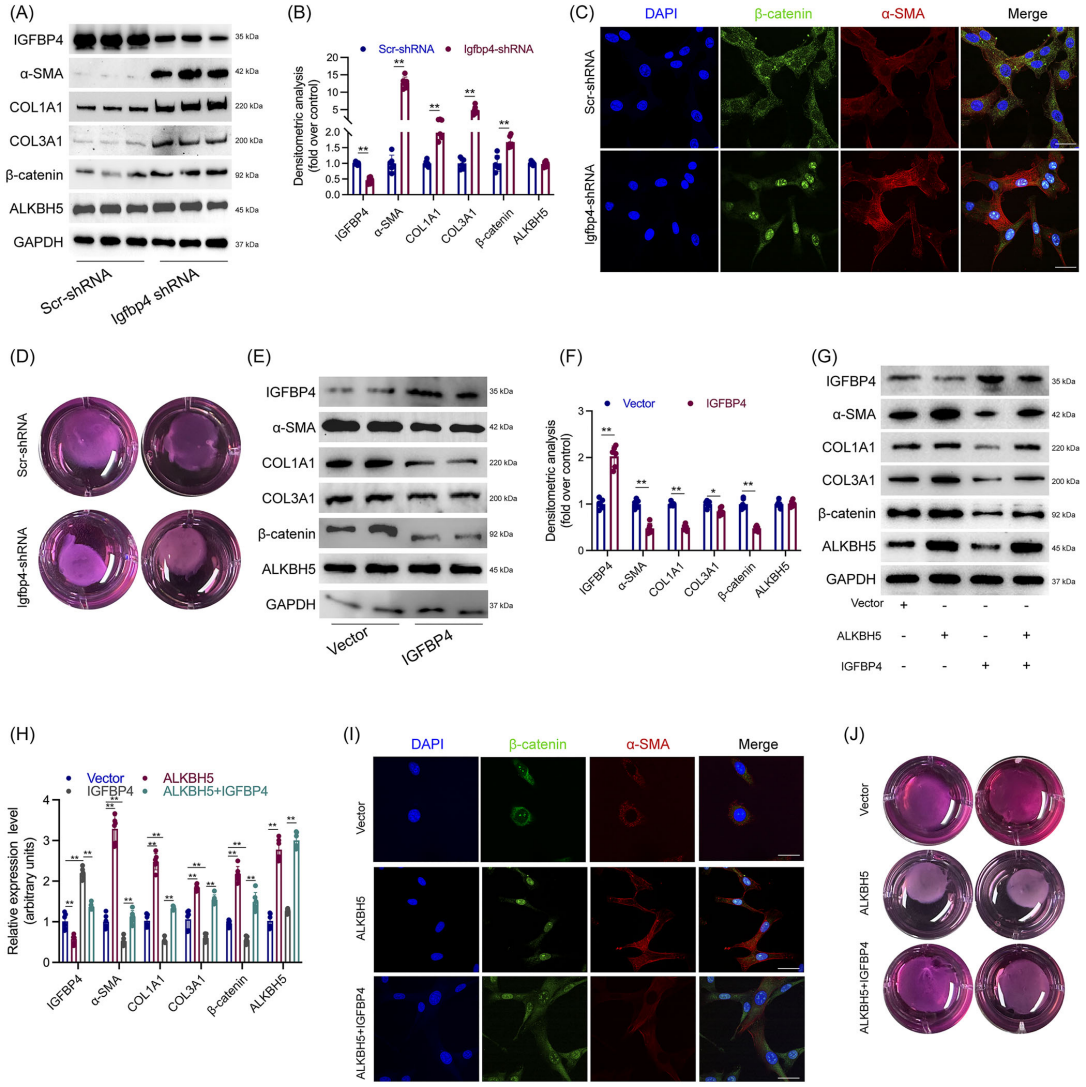
Igfbp4 inhibited fibroblast activation in vitro. (a) Western blot showed the expression of IGFBP4, COL1A1, α‐SMA, AlkB Homologue 5 (ALKBH5), COL3A1 and β‐catenin in cells treated with scr‐shRNA or Igfbp4‐shRNA, and the quantification of the expression levels in western blot were shown in (b). *N* = 4. (c) Immunofluorescence experiment of α‐SMA and β‐catenin in cells treated with scr‐shRNA or Igfbp4‐shRNA. (d) Collagen gel contraction assay showing the contractility of cell treated with scr‐shRNA or Igfbp4‐shRNA. (e) Western blot showed the expression of IGFBP4, COL1A1, α‐SMA, ALKBH5, COL3A1 and β‐catenin in cells treated with Lv‐Igfbp4 or Lv‐vector, and the quantification of the expression levels in western blot were shown in (f). *N* = 4. (g) Western blot showed the expression of IGFBP4, COL1A1, α‐SMA, ALKBH5, COL3A1 and β‐catenin in cells treated with Lv‐vector, Lv‐Alkbh5, Lv‐Igfbp4 or Lv‐Alkbh5 + Lv‐Igfbp4 and the quantification of the expression levels in western blot were shown in (h). *N* = 6. (i) Immunofluorescence experiment of α‐SMA and β‐catenin in cells treated with Lv‐vector, Lv‐Alkbh5 or Lv‐Alkbh5 + Lv‐Igfbp4. (j) Collagen gel contraction assay showing the contractility of cell treated with Lv‐vector, Lv‐Alkbh5 or Lv‐Alkbh5 + Lv‐Igfbp4. Data are presented as mean ± standard deviation (SD); ns: not significant; ^*^ *p* < .05, ^**^ *p* < .01, ^**^ *p* < .001.

Previous studies have reported that IGFBP4 can inhibit fibrosis by suppressing the Wnt/β‐catenin signalling pathway.^20^, ^21^, ^22^ To investigate the functional role of IGFBP4 in fibroblast activation and its regulatory effect on the Wnt/β‐catenin pathway, we isolated fibroblasts from murine laryngotracheal tissue and constructed Igfbp4‐targeting shRNA to knockdown its expression. Knockdown efficiency was confirmed by qPCR and WB (Figure 6a,b). Silencing Igfbp4 significantly increased the expression of fibrotic markers COL1A1, COL3A1, α‐SMA and β‐catenin, as shown by qPCR and WB (Figure 6a,b). Immunofluorescence staining further revealed that Igfbp4 knockdown enhanced β‐catenin expression and nuclear translocation, as well as increased α‐SMA levels (Figure 6c). Moreover, collagen gel contraction assays demonstrated that fibroblast contractility was markedly enhanced following Igfbp4 silencing (Figure 6d).

We next overexpressed IGFBP4 in fibroblasts, and verified overexpression efficiency via qPCR and WB (Figure 6e,f). IGFBP4 overexpression led to a significant reduction in COL1A1, COL3A1, α‐SMA and β‐catenin expression (Figure 6e,f). Notably, when IGFBP4 was overexpressed in fibroblasts already overexpressing ALKBH5, it rescued the upregulation of COL1A1, COL3A1 and α‐SMA induced by ALKBH5 (Figure 6g,h). Immunofluorescence staining further confirmed that the increases in α‐SMA and β‐catenin observed with ALKBH5 overexpression were reversed by IGFBP4 overexpression (Figure 6i). Similarly, the enhanced fibroblast contractility induced by ALKBH5 was effectively blocked by IGFBP4 overexpression, as demonstrated by collagen gel contraction assays (Figure 6j). To further establish the causal relationship, we performed sequential knockdown of ALKBH5 followed by IGFBP4 in fibroblasts in vitro. We found that the suppressed fibrotic phenotype caused by ALKBH5 knockdown was reactivated, as evidenced by restored increases in α‐SMA and ‐COL1A1, COL3A1 expression (Figure S5c,d). Collectively, these findings indicate that IGFBP4 functions downstream of ALKBH5 and suppresses fibroblast activation in vitro, likely through inhibition of the β‐catenin signalling pathway.

### 3.7 Overexpression of IGFBP4 partly alleviated the formation of LTS in vivo

**FIGURE 7:**
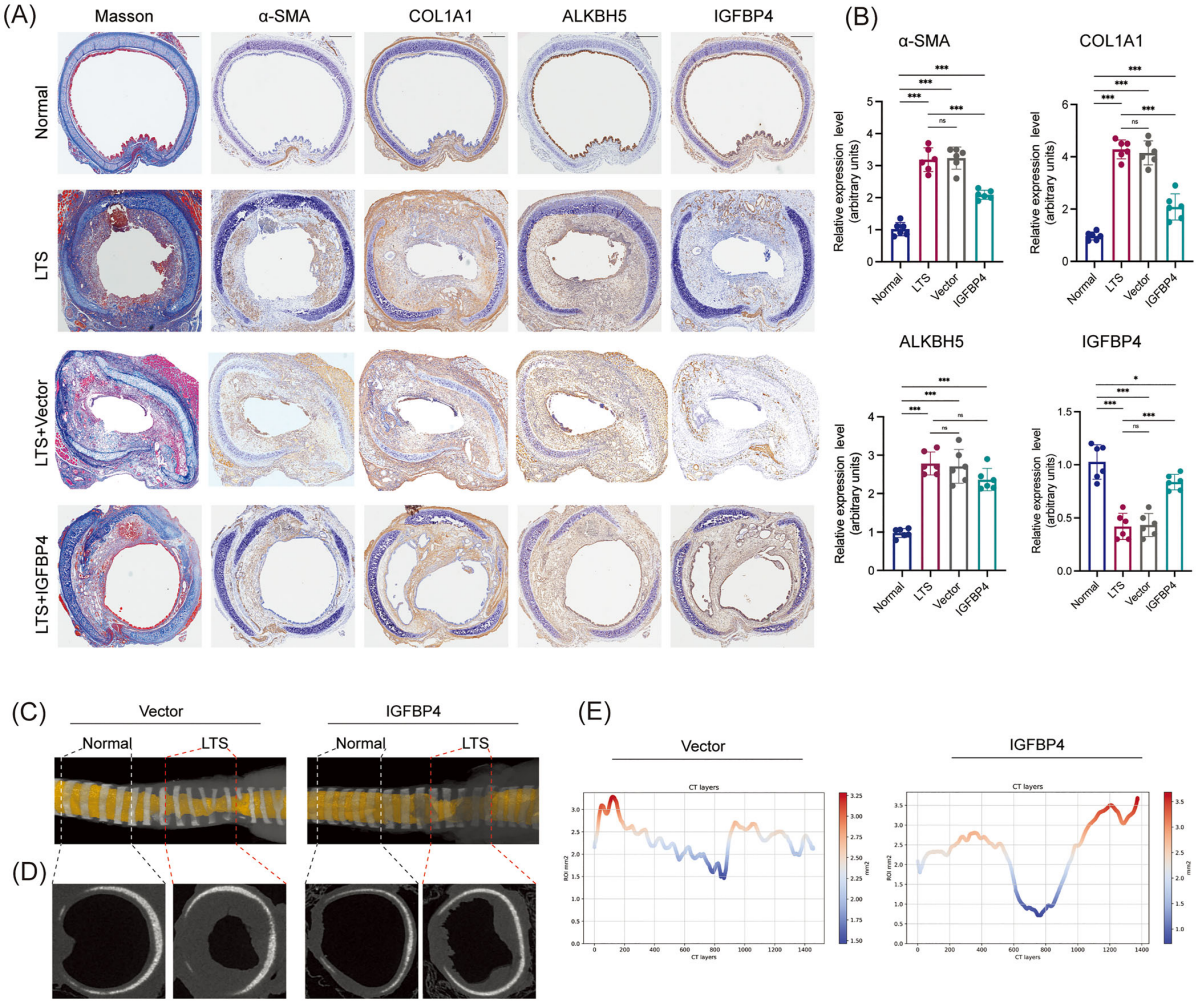
Overexpression of Igfbp4 alleviates the formation of laryngotracheal stenosis (LTS) in vivo. (a) Masson's trichrome staining and immunostaining of COL1A1, α‐SMA, AlkB Homologue 5 (ALKBH5) and IGFBP4 of laryngotracheal tissues from normal rats, LTS rats, AAV‐Igfbp4‐rats and AAV‐vector‐rats at 7 days post‐LTS induction. (b) Quantification of the expression levels of COL1A1, α‐SMA, ALKBH5 and IGFBP4 in immunostaining were shown. *N* = 7. (c) Representative three‐dimensional reconstructed images of laryngotracheal tissues in AAV‐Igfbp4‐rats and AAV‐Ctrl‐rats at 7 days post‐injury. (d) Representative images of thin‐section computed tomography cross‐sections of laryngotracheal tissues in AAV‐Igfbp4‐rats and AAV‐Ctrl‐rats at 7 days post‐injury. (e) Quantification of the lumen diameter from AAV‐Igfbp4‐rats and AAV‐Ctrl‐rats using regions of interest (ROI) at 7 days post‐injury. Data are presented as mean ± standard deviation (SD); ns: not significant; ^*^ *p* < .05, ^**^ *p* < .01, ^**^ *p* < .001.

To investigate the therapeutic potential of IGFBP4 in LTS, we administered Igfbp4 overexpression in LTS‐induced WT rats using an AAV delivery system. AAV‐containing gel was applied around the trachea in both control and experimental groups. Masson's trichrome staining showed that AAV‐Igfbp4 treatment partly reduced collagen deposition compared to control rats and AAV‐vector rats, and the degree of tracheal luminal stenosis was also alleviated. Immunofluorescence staining confirmed increased expression of IGFBP4, accompanied by marked reductions in COL1A1, α‐SMA and ALKBH5 expression in the AAV‐Igfbp4‐treated group (Figure 7a,b). Furthermore, micro‐CT and 3D reconstruction analyses of the LTS tissue revealed that both the narrowed luminal space and inner diameter were markedly improved in the AAV‐Igfbp4 group compared to controls (Figure 7c–e). Taken together, these findings highlight the ALKBH5–IGFBP4 axis as a promising therapeutic target for attenuating fibrosis and alleviating airway obstruction in LTS.

## 4 DISCUSSION

LTS is a pathological condition characterised by the narrowing of the upper airway due to tissue fibrosis and excessive ECM deposition, leading to impaired respiratory function.^23^ It often results from endotracheal intubation and can cause dyspnoea, stridor and respiratory failure.^24^ Emerging evidence indicates that LTS pathogenesis involves multiple interrelated mechanisms at the cellular and molecular levels.^25^ Key mechanisms include immune dysregulation, changes in the local ECM niche, mechanical cues, metabolic alterations and potential involvement of dysbiotic microbiota.^26^ Recent single‐cell transcriptomic studies have revealed significant cellular heterogeneity, identifying distinct fibroblast subpopulations—such as chondrocyte injury‐related fibroblasts and fibrotic fibroblasts—that actively contribute to ECM remodelling. Additionally, epigenetic modifications such as RNA m6A methylation may further modulate fibrotic processes.^17^

Recent studies have revealed that ALKBH5 plays a multifaceted and context‐dependent role in tissue fibrosis. ALKBH5 has cell‐ and organ‐specific regulatory mechanisms that can either aggravate or attenuate fibrotic progression depending on the microenvironmental and pathological conditions. In pulmonary fibrosis, for instance, ALKBH5 promoted fibroblast activation and myofibroblast differentiation via the miR‐320a‐3p/FOXM1 axis, facilitating silica‐induced fibrosis.^27^ However, in other pulmonary contexts, such as radiation‐induced or PM2.5‐triggered fibrosis, ALKBH5 exhibited anti‐fibrotic effects by promoting macrophage autophagy.^28^, ^29^, ^30^ Similarly, ALKBH5 appeared to play divergent roles in liver fibrosis. In non‐alcoholic fatty liver disease, ALKBH5 suppressed hepatic stellate cell activation.^31^ Yet under radiation‐induced liver injury, ALKBH5 promoted fibrosis by demethylating TIRAP mRNA and activating NF‐κB and JNK/Smad2 pathways.^32^ In cardiac fibrosis, ALKBH5 enhanced the stability and expression of ErbB4 mRNA, promoting the transformation of fibroblasts into myofibroblasts and improving fibrosis.^14^ In the condition of AngII‐induced hypertension, ALKBH5 resulted m6A demethylation of IL‐11 mRNA and increased its stability, promoting AngII‐induced macrophage‐to‐myofibroblast transition and cardiac fibrosis.^8^ Even within fibroblasts, ALKBH5's function can vary. It may promote cardiac or pulmonary fibroblast activation by stabilising pro‐fibrotic mRNAs. In a mouse model of silica‐induced pulmonary fibrosis, ALKBH5 was shown to enhance lung fibroblast activation and exacerbate fibrosis, by regulating the miR‐320a‐3p/FOXM1 pathway and/or by directly modulating FOXM1.^27^ However, in hepatic stellate cells, it can inhibit their trans‐differentiation into myofibroblasts depending on the context.^33^ In immune cells, ALKBH5 has been reported to enhance CD4^+^ T‐cell function by modulating m6A‐dependent mRNA stability. Specifically, ALKBH5 reduces m6A modification on IFN‐γ transcripts, thereby stabilising IFN‐γ mRNA and increasing IFN‐γ production. In addition, ALKBH5 regulates the stability of CXCL2 mRNA in CD4^+^ T cells, altering CXCL2 expression and consequently promoting CD4^+^ T‐cell responses and neutrophil recruitment.^7^ For macrophages, multiple studies have indicated that ALKBH5 is involved in regulating macrophage polarisation. In diabetic retinopathy, ALKBH5‐dependent m6A regulation was reported to reduce A20 expression, thereby enhancing M1 polarisation of retinal microglia.^34^ Notably, the effects of ALKBH5 on macrophage polarisation appear to be context dependent. These findings highlight that ALKBH5 does not act as a simple fibrosis driver or suppressor. Instead, it serves as a context‐dependent regulator whose function is determined by the specific cell types, disease models and the m6A‐modified mRNAs involved. Our findings identified the ALKBH5–IGFBP4 axis as a novel regulatory mechanism in LTS, and future studies will be required to determine whether this axis operates in other fibrotic diseases.

While the pro‐fibrotic role of ALKBH5 in fibroblast activation was evident in our in vivo and in vitro models, the downstream molecular mechanism remained to be elucidated. Through integrative transcriptomic (RNA‐seq) and epi‐transcriptomic (MeRIP‐seq) analyses, we identified Igfbp4 as a direct m6A‐modified target of ALKBH5 in fibroblasts. Our results demonstrated that ALKBH5 negatively regulated the mRNA stability of Igfbp4 by removing m6A marks from its 3′‐UTR, leading to decreased Igfbp4 expression. This interaction was further confirmed by RIP and dual‐luciferase reporter assays, which relied on two functional ALKBH5‐binding sites within the Igfbp4 3′‐UTR. ALKBH5 has many target genes. In this study, through MeRIP‐seq and RNA‐seq, we narrowed these down to 18 genes that showed both increased m6A modification and elevated expression levels after ALKBH5 knockdown, including Igfbp4, Obsl1, Scn2a, Nol4l, Tifa, Rfc2, Fzd1, Asb4, Atoh8, Tsen34, Sytl2, Xntrpc,, Agap2, Ckap4, Fbxo48, Mycn and Fbxl15. After conducting a thorough literature review on the functions of these genes, we found that only Igfbp4 might regulate fibrosis‐related genes or signalling pathways. Therefore, we chose Igfbp4 as the target for further investigation. Furthermore, m6A‐mediated regulation of mRNA fate is often executed by reader proteins. In this context, the reduced m6A modification on Igfbp4 transcripts caused by ALKBH5 may weaken the binding of stabilising readers (e.g., the IGF2BP1/2/3 family) or alter recognition by YTH‐domain proteins, thereby affecting Igfbp4 mRNA stability. Identifying the specific reader(s) responsible for Igfbp4 regulation will be an important direction for future investigation to further refine the ALKBH5–IGFBP4 regulatory mechanism.

IGFBP4 is a secreted glycoprotein that regulates cellular behaviour both through IGF‐dependent and IGF‐independent mechanisms. Canonically, IGFBP4 modulates the bioavailability and signalling activity of IGF‐I and IGF‐II, thereby influencing cell proliferation, differentiation, apoptosis and tissue remodelling across various physiological contexts.^35^, ^36^ However, emerging evidence has expanded our understanding of IGFBP4's non‐canonical roles. IGFBP4 can directly interact with low‐density lipoprotein receptor‐related proteins 5 and 6 (LRP5/6), functioning as a negative regulator of the Wnt/β‐catenin signalling pathway.^37^, ^38^ This function has been implicated in regulating processes such as cardiogenesis, neural stem cell differentiation and tumourigenesis.^39^, ^40^ Additionally, IGFBP4 can be packaged into exosomes, serving as a paracrine mediator in the tumour microenvironment. For instance, exosomal IGFBP4 derived from hepatitis C virus (HCV)‐infected hepatocytes was shown to promote cell migration and proliferation, contributing to hepatocellular carcinoma progression.^41^ These multifaceted roles underscore the context‐dependent nature of IGFBP4, which may exert either inhibitory or promotive effects on tissue remodelling and disease pathogenesis depending on the cellular niche and upstream regulatory cues.

In the context of fibrosis, previous studies have reported that IGFBP4 exerts anti‐fibrotic effects by blocking TGF‐β‐induced ECM production and reducing collagen deposition in bleomycin‐induced pulmonary fibrosis models.^20^ Although the molecular details of these effects were not fully characterised, these observations suggest that IGFBP4 acts as an inhibitor of fibroblast activation and myofibroblast transition. Our findings are consistent with and extend this finding. We observed that IGFBP4 expression was significantly downregulated during LTS progression, coinciding with elevated ALKBH5 expression. IGFBP4 overexpression in LTS animal models mitigated collagen deposition and improved airway lumen patency, further supporting its protective role. These findings establish IGFBP4 as a critical functional mediator downstream of ALKBH5, acting to restrain pathological fibroblast activation and tissue fibrosis.

## 5 CONCLUSIONS

Our study demonstrated that ALKBH5 played a critical role in airway fibrosis through the regulation of Igfbp4 mRNA at post‐transcriptional level via m6A‐dependent mechanism. We identified that Igfbp4 was a direct target of ALKBH5 in LTS. ALKBH5 could reduce Igfbp4 mRNA stability in fibroblasts of LTS, leading to β‐catenin activation and overexpression of IGFBP4 could partly alleviate ALKBH5 up‐regulated fibroblast activation. Our work revealed a previously unrecognised signalling axis involving ALKBH5‐IGFBP4 in fibroblast activation. Targeting ALKBH5 or supplementing IGFBP4 may become novel therapeutic strategies for LTS.

## AUTHOR CONTRIBUTIONS

Jing Wang designed the methodology; carried out the animal models and parts of cellular investigation, and wrote the original draft and revised the manuscript. Ziwei Liao conceived the study; carried out the cellular and molecular investigation, and wrote the original draft. Mengrou Xu revised and edited the manuscript. Bin Hu and Yangyang Zheng carried out the cellular investigation. Qianhui Xia prepared the visualisations. Hongming Xu supervised the work. All the authors reviewed the manuscript.

## CONFLICT OF INTEREST STATEMENT

The authors declare they have no conflicts of interest.

## ETHICS STATEMENT

All animal experiments were conducted in accordance with the guidelines of the Institutional Animal Care and Use Committee of Shanghai Children's Hospital (2021RY024‐E01).

## Supporting information



Supporting information

Supporting information

## Data Availability

RNA‐seq and MeRIP‐seq data have been deposited in the NCBI Sequence Read Archive (SRA) database with the accession number PRJNA1311743. Single‐cell RNA sequencing data have been deposited in the NCBI SRA database with the accession number PRJNA1221101.
