# Supplementary material for: M6A demethylase ALKBH5 mediated Igfbp4 mRNA m6A modification drives fibroblast activation and pathological upper airway fibrosis
Source: Clin Transl Med. 2026 Apr 21;16(4):e70656. doi: 10.1002/ctm2.70656 (PMC13100485; doi:10.1002/ctm2.70656)
Supplement: Supplementary file 2 — Supporting information [file CTM2-16-e70656-s001.docx]

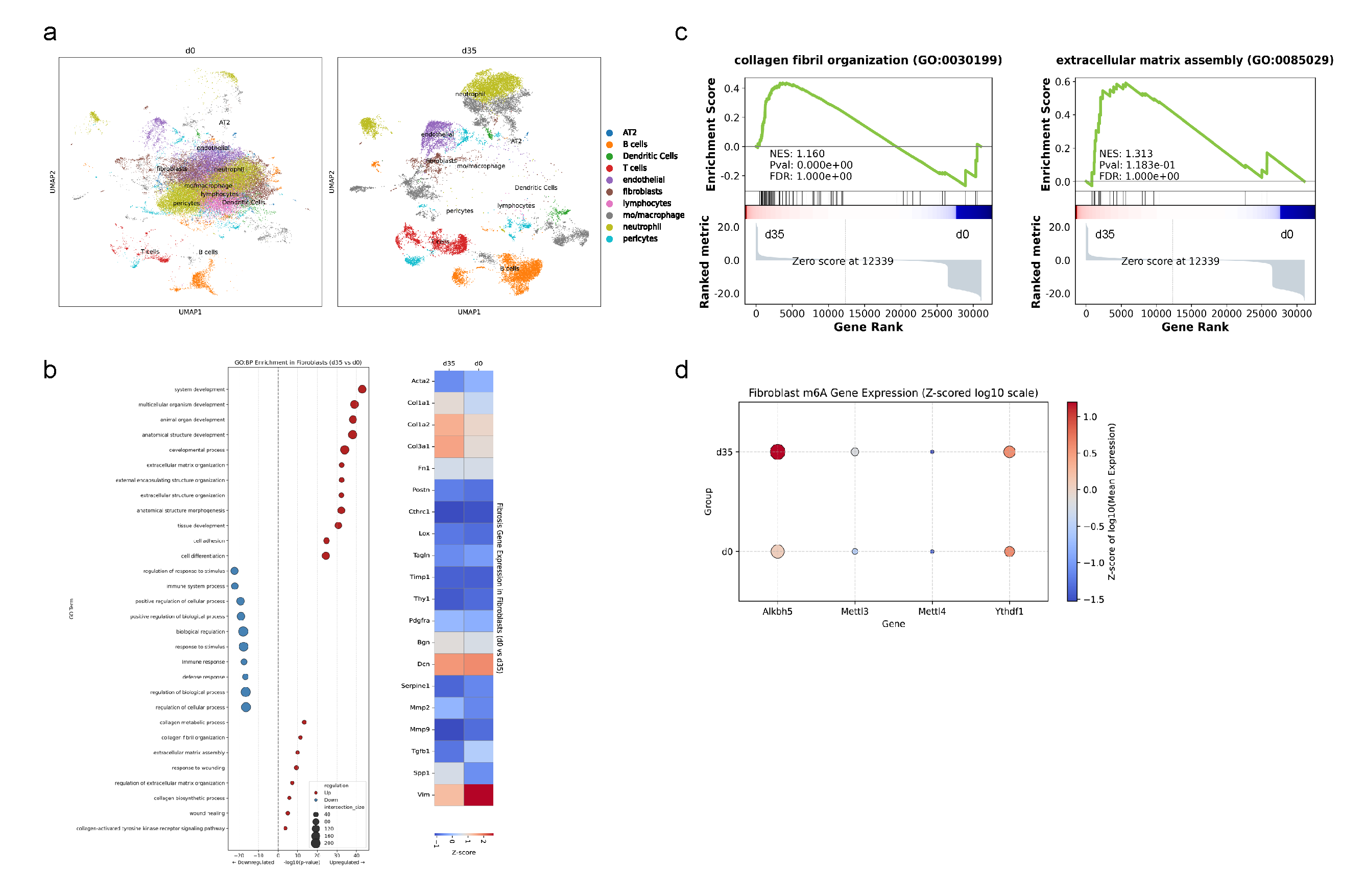
**Supplementary figures**

**Supplementary figure 1. single-cell RNA sequencing data from pulmonary fibrosis model.** (a) Manifold Approximation and Projection (UMAP) depicting major cell types identified in normal and pulmonary fibrosis. (b) GO enrichment scatterplot displayed the top significantly enriched biological processes identified in fibroblasts in pulmonary fibrosis. Heatmap displayed the top significantly changed genes. (c) GSEA displayed the top significantly enriched gene sets in fibroblasts in pulmonary fibrosis. (d) Scatterplot displayed the expression in of Mettl3, Mettl4, Ythdf1, and Alkbh5 in fibroblasts in pulmonary fibrosis. Mouse lung scRNA-seq: 3 samples, GEO GSE250396, https://www.ncbi.nlm.nih.gov/geo/query/acc.cgi?acc=GSE250396.


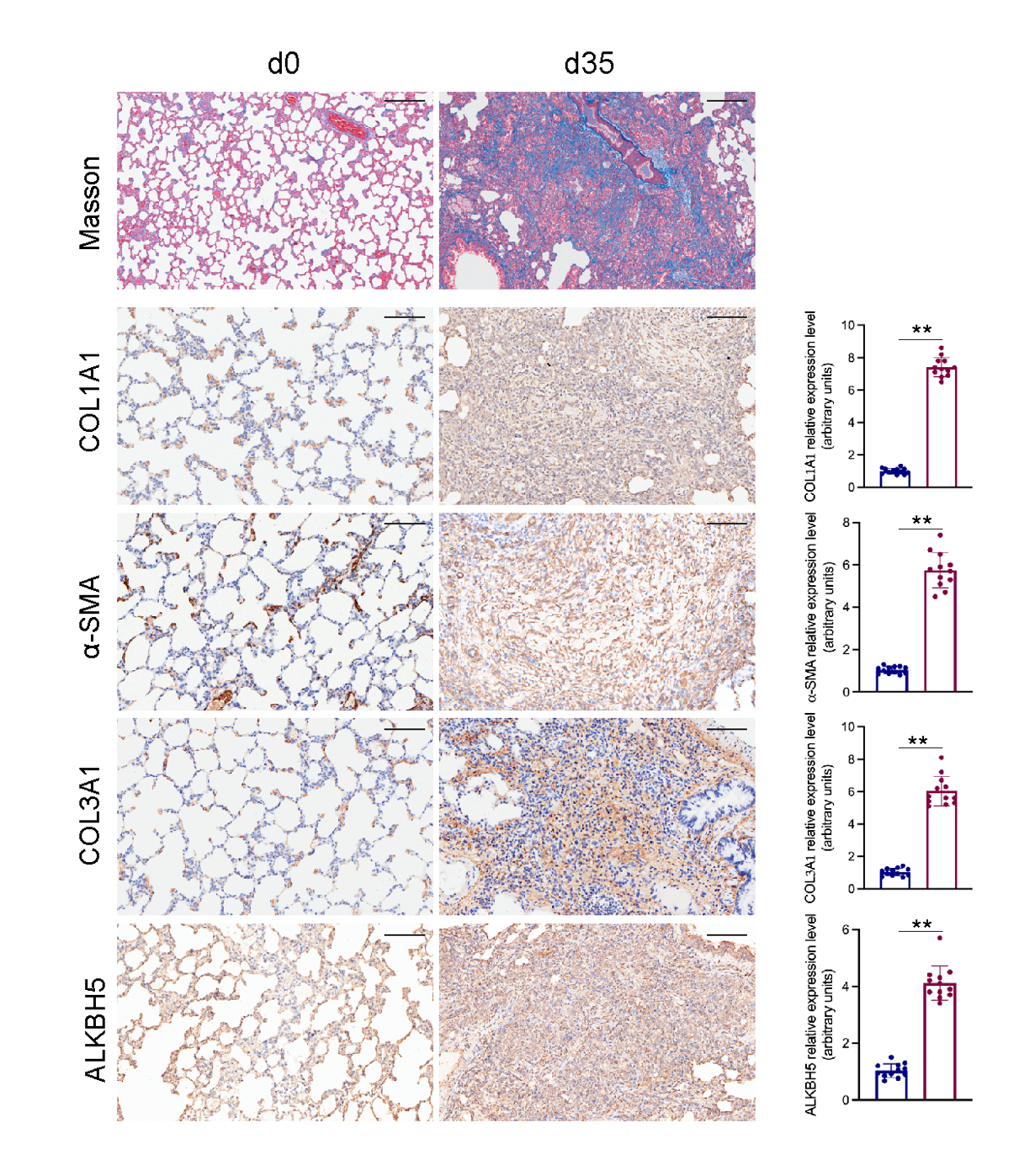


**Supplementary figure 2. The expressions of fibrotic markers in the rat models of pulmonary fibrosis.** Masson’s trichrome staining and immunostaining of COL1A1, COL3A1, α-SMA and ALKBH5 of lung tissues in normal (d0) and pulmonary fibrosis (d35). Quantification of the immunostaining were shown on the right. Data are presented as mean ± SD; **P < 0.001.


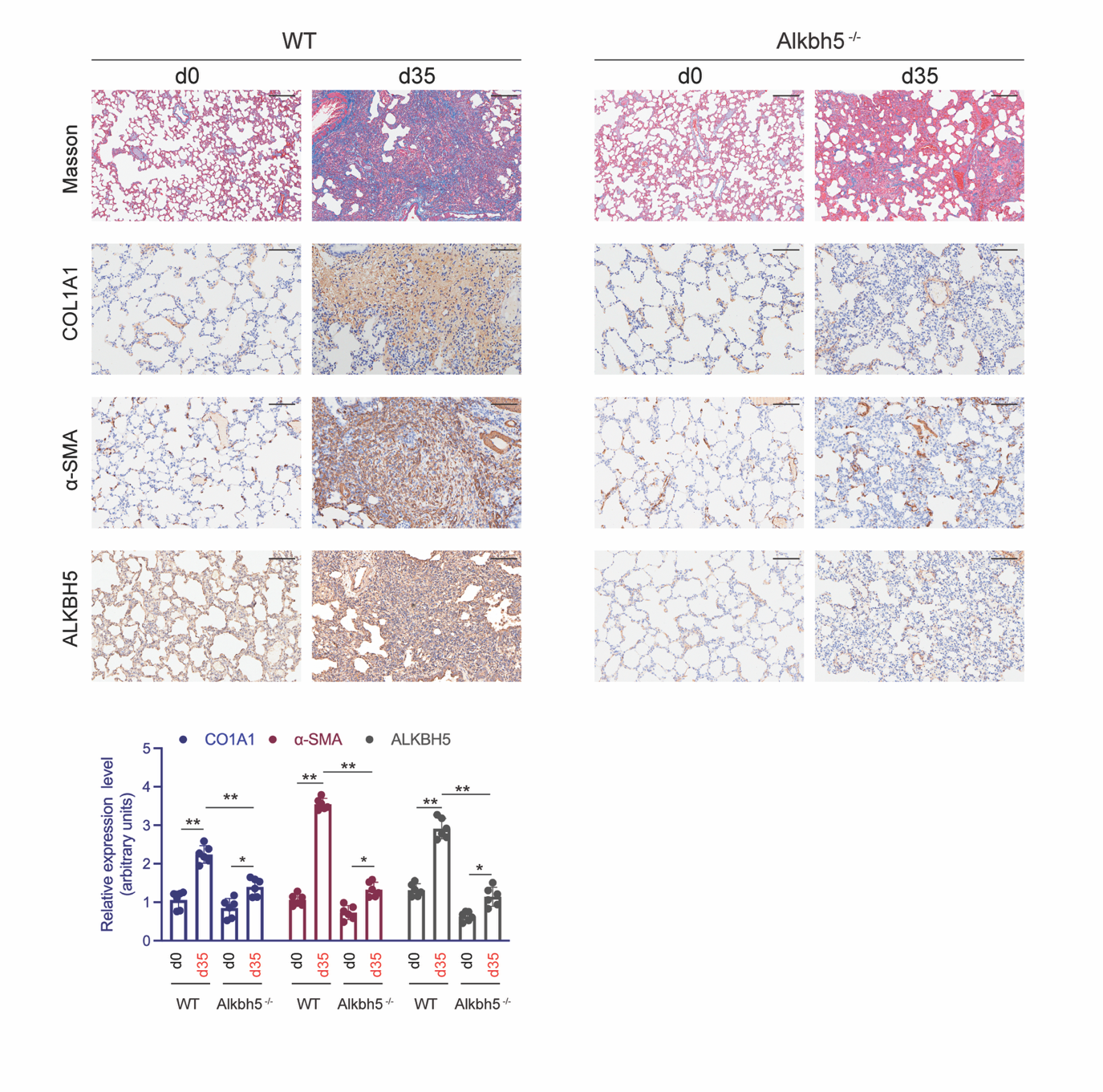


**Supplementary figure 3. The expressions of fibrotic markers in pulmonary fibrosis models in WT and Alkbh5⁻/⁻ rats.** Masson’s trichrome staining and immunostaining of COL1A1, α-SMA and ALKBH5 of lung tissues in normal (d0) and pulmonary fibrosis (d35) in WT and Alkbh5⁻/⁻ rats. Quantification of the immunostaining were shown in below. Data are presented as mean ± SD; *P < 0.01; **P < 0.001.


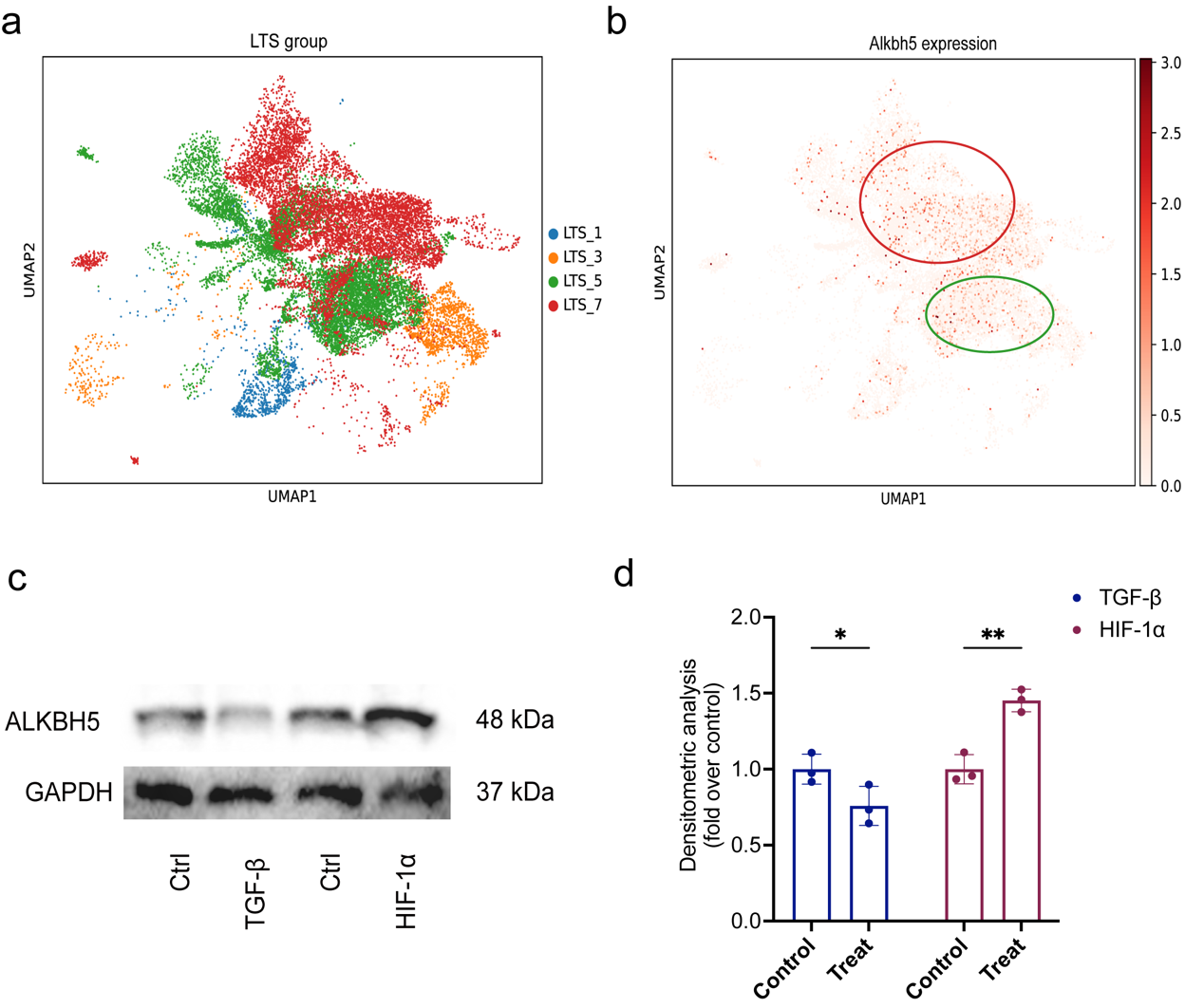


**Supplementary figure 4. Single-cell analysis and validation of ALKBH5 expression under HIF-1α and TGF-β stimulation.** (a) UMAP maps displayed the distribution of cells at different time points. Distinct time points were annotated by different colors. (b) UMAP maps displayed the distribution of ALKBH5 expression across all cells. (c) Western blot showed the expression of ALKBH5 and GAPDH in in fibroblasts treated with HIF-1α or TGF-β. The expression levels were quantified on figure. (d). Data are presented as mean ± standard deviations (SD); ns: not significant; *P < 0.01; **P < 0.001.


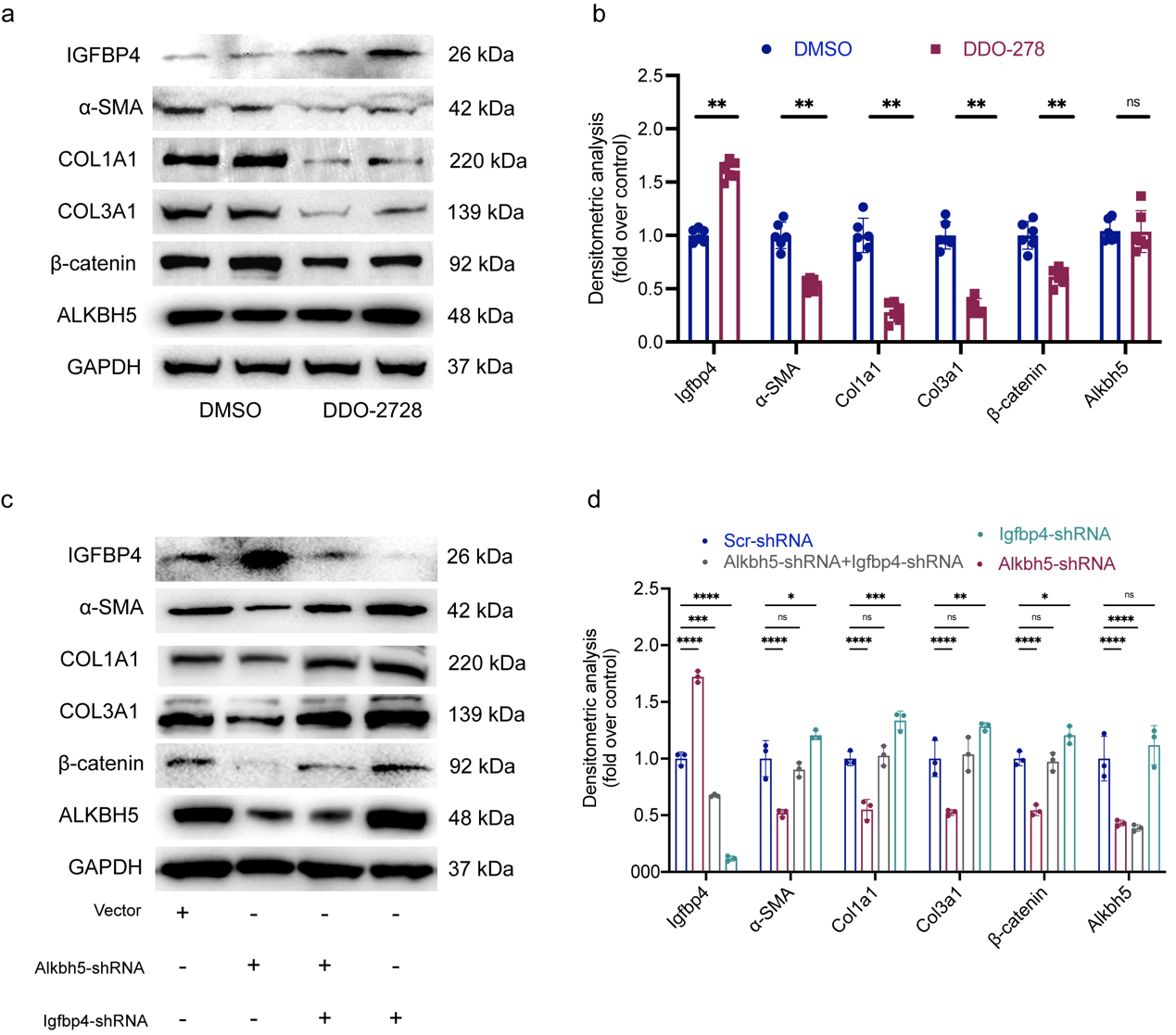


**Supplementary figure 5. ALKBH5 inhibition and IGFBP4 knockdown modulate fibrosis-related protein expression.** (a) Western blot showed the expression of IGFBP4, COL1A1, α-SMA, ALKBH5, COL3A1, and β-catenin in cells treated with DDO-2728(selective ALKBH5 inhibitor) or DMSO , and the quantification of the expression levels in western blot were shown in (b). N=6. (c) Western blot showed the expression of IGFBP4, COL1A1, α-SMA, ALKBH5, COL3A1, and β-catenin in cells treated with scr-shRNA, Igfbp4-shRNA, Alkbh5-shRNA or gfbp4-shRNA+ Alkbh5-shRNA, and the quantification of the expression levels in western blot were shown in (d). N=3. Data are presented as mean ± standard deviations (SD); ns: not significant; *P < 0.01; **P < 0.001.
